# Maximum Somatic Allele Frequency in Combination With Blood-Based Tumor Mutational Burden to Predict the Efficacy of Atezolizumab in Advanced Non-small Cell Lung Cancer: A Pooled Analysis of the Randomized POPLAR and OAK Studies

**DOI:** 10.3389/fonc.2019.01432

**Published:** 2019-12-17

**Authors:** Yu-tong Chen, Sharvesh Raj Seeruttun, Xiang-yuan Wu, Zi-xian Wang

**Affiliations:** ^1^Department of Medical Oncology, Third Affiliated Hospital of Sun Yat-sen University, Guangzhou, China; ^2^Department of Surgical Oncology, Sun Yat-sen University Cancer Center, State Key Laboratory of Oncology in South China, Collaborative Innovation Center for Cancer Medicine, Guangzhou, China; ^3^Department of Medical Oncology, Sun Yat-sen University Cancer Center, State Key Laboratory of Oncology in South China, Collaborative Innovation Center for Cancer Medicine, Guangzhou, China

**Keywords:** maximum somatic allele frequency (MSAF), blood-based tumor mutational burden (bTMB), atezolizumab, docetaxel, non-small cell lung cancer (NSCLC)

## Abstract

**Background:** Blood-based tumor mutational burden (bTMB) was recently found to be suboptimal in predicting overall survival (OS) benefits of atezolizumab over docetaxel among patients with advanced non-small cell lung cancer (NSCLC). The maximum somatic allele frequency (MSAF) is an indicator of the proportion of tumor-derived plasma DNA, which could affect the concordance between bTMB and tissue-based TMB. Therefore, we aimed to evaluate the utility of MSAF, alone or in combination with bTMB, to identify NSCLC patients with or without survival benefit from atezolizumab over docetaxel.

**Methods:** We analyzed the individual patient-level data from the randomized POPLAR and OAK studies. The bTMB and MSAF were derived from the pre-treatment blood-based genomic data.

**Results:** In both the bTMB-high (i.e., bTMB ≥ 13) and bTMB-low subgroups, atezolizumab significantly improved OS compared with docetaxel (hazard ratio [HR] = 0.43 [95% CI, 0.29–0.65], *P* < 0.001 and HR = 0.73 [95% CI, 0.61–0.87], *P* < 0.001, respectively). Among patients with a low MSAF (i.e., MSAF < 10.3%), OS significantly favored atezolizumab (HR = 0.59 [95% CI, 0.48–0.72], *P* < 0.001), whereas OS with atezolizumab was similar to that with docetaxel in the MSAF-high subgroup (HR = 0.91 [95% CI, 0.68–1.20], *P* = 0.500; interaction test *P* = 0.017). Among patients from the bTMB-low and MSAF-high subgroup, OS was numerically worse with atezolizumab than with docetaxel (HR = 1.06 [95% CI, 0.78–1.45], *P* = 0.710); in contrast, OS was significantly improved with atezolizumab compared with docetaxel in those with either a high bTMB or low MSAF (HR = 0.57 [95% CI, 0.47–0.69], *P* < 0.001; interaction test *P* < 0.001). Consistent findings were obtained for progression-free survival data.

**Conclusions:** MSAF alone or in combination with bTMB can effectively distinguish patients with or without survival benefit from atezolizumab compared with docetaxel. MSAF and the combined bTMB-MSAF classification may become practical predictive markers for atezolizumab in advanced NSCLC.

## Introduction

Tumor mutational burden (TMB) is being extensively studied as a promising biomarker for predicting the efficacy of immune checkpoint inhibitors (ICIs) (1–3). Mounting data have found that a high tissue-based TMB (tTMB) was associated with greater clinical benefit from anti-PD-1 therapies in patients with advanced non-small cell lung cancer (NSCLC) (4–7). Additionally, there has been an increasing interest in exploring the blood-based TMB (bTMB) as a non-invasive predictive marker for ICI therapies (8, 9). In a recent study on advanced NSCLC, although bTMB was found predictive of progression-free survival (PFS) advantage of atezolizumab over docetaxel, it failed to predict overall survival (OS) benefits from atezolizumab (8).

The maximum somatic allele frequency (MSAF) is a useful bioinformatics tool for estimating the amount of tumor fraction of cell-free DNA in peripheral blood samples (10). Previous studies have revealed that a lower MSAF level was associated with a higher risk of missing important genomic alterations in the plasma, such as EGFR exon 19 deletion and EGFR T790M that are predictive of response to EGFR tyrosine kinases in advanced NSCLC (10, 11). However, there exist limited data regarding the association between MSAF and treatment outcomes from ICIs. In the recent B-F1SRT study on atezolizumab in advanced NSCLC, a MSAF <1% was associated with a higher response rate and better PFS, but this effect was dependent on baseline tumor burden (12).

It has been suggested that a low MSAF could contribute to a poorer consistency between bTMB and tTMB (8). For instance, a low MSAF could lead to a lower detection rate of tumor somatic mutations in blood samples (8), in which case a fraction of tTMB-high patients could be misclassified as bTMB-low. Therefore, we hypothesized that incorporating MSAF with bTMB can partially lower the risk of misclassifying tTMB-high cases as bTMB-low, and thus mitigate the discordance between bTMB and tTMB and improve the differentiation between patients with or without survival benefits from ICIs.

In this pooled analysis of the randomized POPLAR (NCT01903993) and OAK (NCT02008227) studies (13, 14), we comprehensively investigated the performance of MSAF alone or in combination with bTMB in predicting the comparative efficacy of atezolizumab and docetaxel among patients with advanced NSCLC.

## Materials and Methods

We included clinical data and pre-treatment blood-based genomic data from patient cohorts from the POPLAR (NCT01903993) and OAK (NCT02008227) studies (8). Briefly, the POPLAR trial was a randomized phase 2 study and the OAK trial a randomized phase 3 study, both comparing second- or third-line atezolizumab with docetaxel in patients with advanced NSCLC (intention-to-treat population *N* = 287 and *N* = 850, respectively) (13, 14). The POPLAR trial was a randomized phase 2 study and the OAK trial a randomized phase 3 study, both comparing second- or third-line atezolizumab with docetaxel in patients with advanced NSCLC (intention-to-treat population *N* = 287 and *N* = 850, respectively) (13, 14). As described by Gandara et al. (8), 853 patients (211 from POPLAR and 642 from OAK) with available bTMB and MSAF data after quality control were eligible for our study. As previously described (8), the MSAF was measured as the highest allele fraction for confirmed somatic base substitutions, and the bTMB was calculated by counting all single-nucleotide variants with allele frequencies of ≥0.5%, excluding driver mutations. According to recent findings from Wang et al. (15), mutations with allele frequencies of >5% were also filtered out in calculation of bTMB in order to weaken the correlation between bTMB and MSAF.

OS was defined as the time from the date of randomization to the date of death from any cause. PFS was defined as the time from the date of randomization to the date of disease progression (per RECIST v1.1) or death from any cause, whichever occurred first. OS and PFS hazard ratios (HRs) and corresponding 95% confidence intervals (CIs) were estimated using multivariable Cox proportional hazards models adjusted for baseline covariates (i.e., age, sex, race, performance status, histology, number of metastatic sites, number of prior therapies, and tobacco use history).

To identify the optimal cutoff values for bTMB (or MSAF), we constructed multivariable Cox models including bTMB (or MSAF), treatment group, and their interaction term, adjusted for the aforementioned baseline covariates. We then applied the Chow test (16) to determine the structural breakpoint for the curve depicting the association between the cutoff for bTMB (or MSAF) and the standardized magnitude of interaction (measured as the wald test Z score, i.e., the coefficient of the interaction term divided by its standard error) between bTMB (or MSAF) and treatment group. The structural breakpoint was considered the threshold of clinico-biological impact.

The predictive accuracy of prognostic models was investigated using a time-dependent ROC analysis. A two-sided *P* < 0.05 was considered statistically significant. All statistical analyses were performed using R v. 3.5.1 (http://www.r-project.org).

## Results

### bTMB Failed to Identify Patients Who Did Not Benefit From Atezolizumab

**Figure 1:**
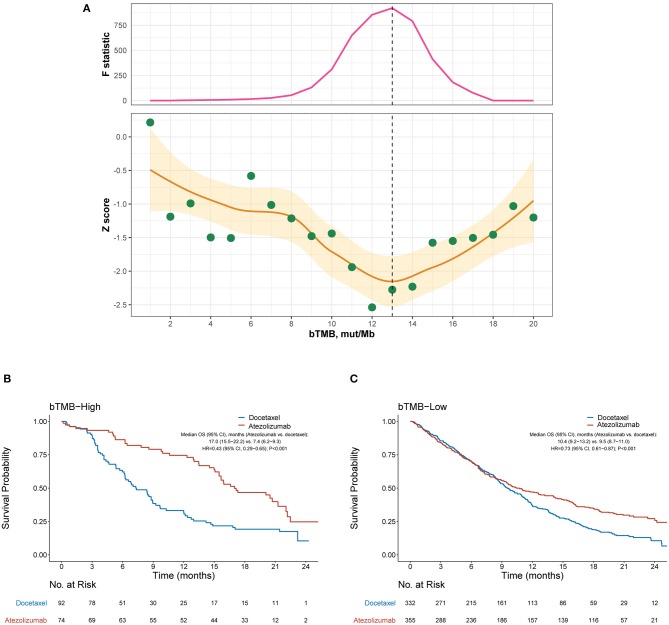
Overall survival outcomes from atezolizumab vs. docetaxel in the bTMB-high and bTMB-low subgroups. **(A)** Identification of the optimal cutoff for bTMB based on Chow test. The standardized interaction effect (measured as the wald test Z score, i.e., the coefficient of the interaction between bTMB and treatment divided by its standard error), as well as the Chow F statistic, are plotted vs. the examined cutoff for bTMB. A Z score <0 indicates that the treatment effect was more prominent in bTMB-high cases, and a lower Z score indicates stronger evidence for superior efficacy of atezolizumab to docetaxel in bTMB-high cases. The yellow curve and ribbon denote the LOESS smoother and its standard error. A structural breakpoint of 13 that maximized the Chow F statistic (i.e., minimizing the ordinary least squares estimator) was identified by Chow test. **(B,C)** Overall survival with atezolizumab vs. docetaxel in the bTMB-high (i.e., bTMB ≥ 13) subgroup and bTMB-low (i.e., bTMB < 13) subgroup, respectively. HRs were adjusted for age, sex, race, performance status, histology, number of metastatic sites, number of prior therapies, and tobacco use history. bTMB, blood-based tumor mutational burden; HR, hazard ratio; CI, confidence interval.

As both the POPLAR and OAK studies demonstrated significantly improved OS with atezolizumab (13, 14), we focused on the identification of patients who did not benefit from atezolizumab. Figure 1A depicts how the interaction between bTMB and the efficacy of atezolizumab vs. docetaxel differed according to the cutoff for bTMB. Overall, the treatment effect was more prominent in bTMB-high cases, as indicated by negative Z scores. The interaction between bTMB and treatment effect on OS tended to augment when the bTMB cutoff increased at first, but later showed a reverse pattern when the bTMB cutoff exceeded certain values. Based on the Chow test, a bTMB cutoff of 13 was identified as the breakpoint and was used to define bTMB-high and bTMB-low cases in subsequent analyses. Notably, atezolizumab significantly improved OS compared with docetaxel in both the bTMB-high (HR, 0.43 [95% CI, 0.29–0.65], *P* < 0.001; Figure 1B) and bTMB-low (HR, 0.73 [95% CI, 0.61–0.87], *P* < 0.001; interaction test *P* = 0.023; Figure 1C) subgroups.

### High MSAF Was Associated With Minimal Benefit of Atezolizumab

**Figure 2:**
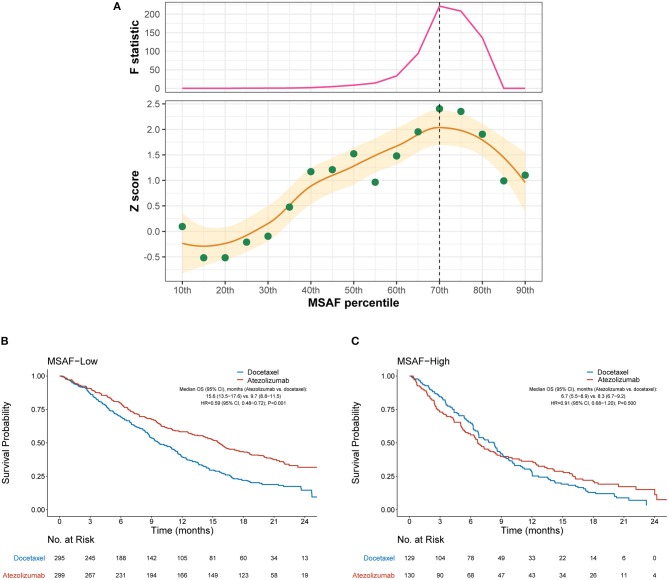
Overall survival outcomes from atezolizumab vs. docetaxel in the MSAF-high and MSAF-low subgroups. **(A)** Identification of the optimal cutoff for MSAF based on Chow test. The standardized interaction effect (measured as the wald test Z score, i.e., the coefficient of the interaction between MSAF and treatment divided by its standard error), as well as the Chow F statistic, are plotted vs. the examined cutoff for MSAF. A Z score >0 indicates that the treatment effect was less prominent in MSAF-high cases, and a higher Z score indicates stronger evidence for superior efficacy of atezolizumab to docetaxel in MSAF-low cases. The yellow curve and ribbon denote the LOESS smoother and its standard error. A structural breakpoint of the 70th percentile (MSAF = 10.3%) that maximized the Chow F statistic (i.e., minimizing the ordinary least squares estimator) was identified by Chow test. **(B,C)** Overall survival with atezolizumab vs. docetaxel in the MSAF-low (i.e., MSAF < 10.3%) subgroup and MSAF-high (i.e., MSAF ≥ 10.3%) subgroup, respectively. HRs were adjusted for age, sex, race, performance status, histology, number of metastatic sites, number of prior therapies, and tobacco use history. MSAF, maximum somatic allele frequency; HR, hazard ratio; CI, confidence interval.

We then evaluated the efficacy of atezolizumab vs. docetaxel in MSAF-high and MSAF-low cases defined by various cut-points of MSAF. Figure 2A illustrates how the interaction between MSAF and the efficacy of atezolizumab vs. docetaxel varied by the cutoff for MSAF. Overall, the treatment effect attenuated in MSAF-high cases, as indicated by positive Z scores. The interaction between MSAF and treatment effect on OS tended to augment when the MSAF cutoff increased at first, but later showed a reverse pattern when the MSAF cutoff exceeded certain values. Based on the Chow test, a MSAF cutoff of 10.3% (70th percentile) was identified as the breakpoint and was used to define MSAF-high and MSAF-low cases in subsequent analyses. Among the MSAF-low subgroup, OS was significantly improved with atezolizumab compared with docetaxel (HR, 0.59 [95% CI, 0.48–0.72], *P* < 0.001; Figure 2B), whereas OS with atezolizumab was similar to that with docetaxel in the MSAF-high subgroup (HR, 0.91 [95% CI, 0.68–1.20], *P* = 0.500; interaction test *P* = 0.017; Figure 2C). After adjusted for baseline covariates including the number of metastatic sites, a high MSAF was associated with worse OS in both treatment arms, but this effect was more prominent in the atezolizumab arm (Supplementary Figure 1).

### Combination of bTMB and MSAF Further Improved the Predictive Accuracy for Benefit of Atezolizumab

**Figure 3:**
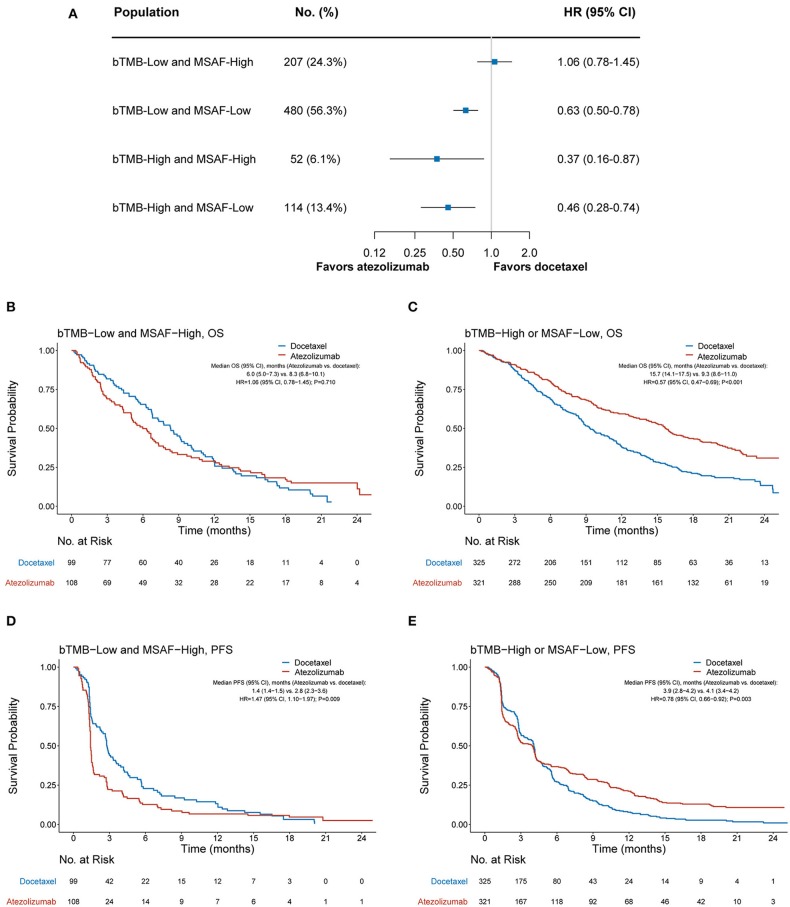
Overall survival outcomes from atezolizumab vs. docetaxel according to the combined bTMB-MSAF status. **(A)** The comparative efficacy of atezolizumab vs. docetaxel regarding overall survival among bTMB-low (i.e., bTMB < 13) and MSAF-high (i.e., MSAF ≥ 10.3%), bTMB-low and MSAF-low, the bTMB-high and MSAF-high, and bTMB-high and MSAF-low cases. **(B,C)** Overall survival with atezolizumab vs. docetaxel in the bTMB-low and MSAF-high subgroup and the bTMB-high or MSAF-low subgroup, respectively. **(D,E)** Progression-free survival with atezolizumab vs. docetaxel in the bTMB-low and MSAF-high subgroup and the bTMB-high or MSAF-low subgroup. HRs were adjusted for age, sex, race, performance status, histology, number of prior therapies, and tobacco use history. bTMB, blood-based tumor mutational burden; MSAF, maximum somatic allele frequency; HR, hazard ratio; CI, confidence interval.

We further investigated whether the combination of bTMB and MSAF would achieve an improved performance in differentiating patients with or without OS benefit from atezolizumab. MSAF showed statistically significant but numerically minimal correlation with bTMB (Pearson's correlation coefficient, 0.09; *P* = 0.009). OS was found significantly better with atezolizumab than with docetaxel in the bTMB-low and MSAF-low subgroup (HR, 0.63 [95% CI, 0.50–0.78], *P* < 0.001), the bTMB-high and MSAF-high subgroup (HR, 0.37 [95% CI, 0.16–0.78], *P* = 0.023), and the bTMB-high and MSAF-low subgroup (HR, 0.46 [95% CI, 0.28–0.74], *P* = 0.002; Figure 3A). Therefore, we combined these subgroups into the “bTMB-high or MSAF-low” subgroup for subsequent analyses. Patient characteristics were balanced between treatment arms in the bTMB-low and MSAF-high subgroup (207 cases, 24.3%) and the bTMB-high or MSAF-low subgroup (646 cases, 75.7%; Supplementary Table 1).

Among the bTMB-low and MSAF-high subgroup, OS was numerically worse with atezolizumab (median OS, 6.0 months [95% CI, 5.0–7.3]) than with docetaxel (median OS, 8.3 months [95% CI, 6.8–10.1]; HR, 1.06 [95% CI, 0.78–1.45], *P* = 0.710; Figure 3B). In contrast, OS was significantly improved with atezolizumab compared with docetaxel in the bTMB-high or MSAF-low subgroup (median OS, 15.7 months [95% CI, 14.1–17.5] vs. 9.3 months [95% CI, 8.6–11.0]; HR, 0.57 [95% CI, 0.47–0.69], *P* < 0.001; Figure 3C). Notably, the interaction test between treatment group and the combined bTMB-MSAF classification (i.e., “bTMB-low and MSAF-high” vs. “bTMB-high or MSAF-low”) yielded a higher statistical significance level than that between treatment group and MSAF (*P*_*interation*_ <0.001 vs. *P*_*interation*_ = 0.017), along with a larger HR in the bTMB-low and MSAF-high than in the MSAF-high subgroup (1.06 vs. 0.91; Figures 2B, 3B). Meanwhile, a concurrent bTMB-low and MSAF-high status was significantly associated with a dismal OS in the atezolizumab arm (HR, 2.21 [95% CI, 1.70–2.86], *P* < 0.001) but not the docetaxel arm (HR, 1.27 [95% CI, 0.99–1.64], *P* = 0.057; Supplementary Figure 2).

The combined bTMB-MSAF classification can also identify patients who had no PFS benefit from atezolizumab. Among the bTMB-low and MSAF-high subgroup, PFS was significantly worse with atezolizumab (median PFS, 1.4 months [95% CI, 1.4–1.5]) than with docetaxel (median PFS, 2.8 months [95% CI, 2.3–3.6]; HR, 1.47 [95% CI, 1.10–1.97], *P* = 0.009; Figure 3D), whereas PFS significantly favored atezolizumab in the bTMB-high or MSAF-low subgroup (median PFS, 3.9 months [95% CI, 2.8–4.2] vs. 4.1 months [95% CI, 3.4–4.2]; HR, 0.78 [95% CI, 0.66–0.92], *P* = 0.003; Figure 3E; interaction test *P* < 0.001).

As shown in Supplementary Figure 3, receiver operating characteristic (ROC) analyses further indicated the superior accuracy of the combined bTMB-MSAF classification, as compared with that of bTMB or MSAF alone, for prediction of OS and PFS in patients treated with atezolizumab.

### Sensitivity Analyses

**Figure 4:**
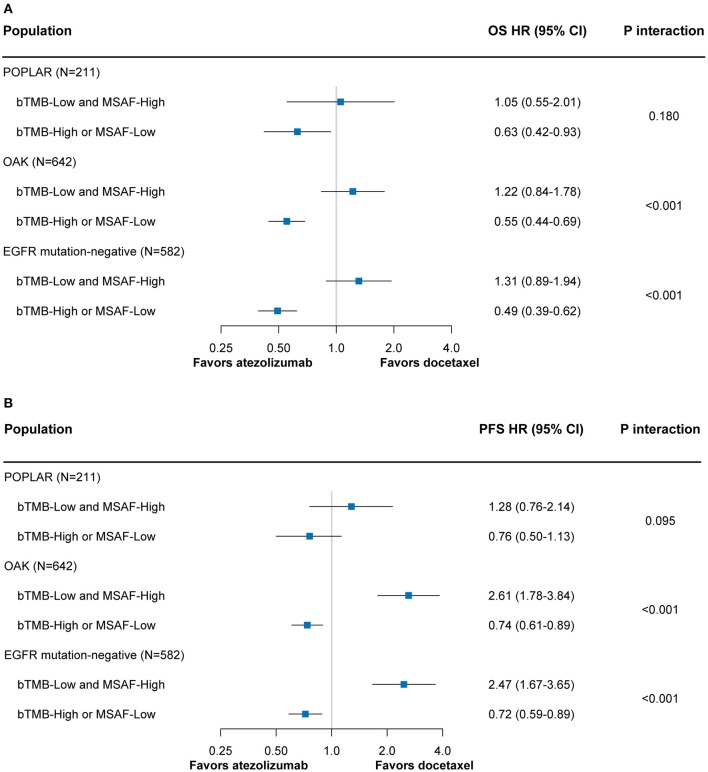
The predictive value of the bTMB-MSAF classification for OS **(A)** and PFS **(B)** outcomes from atezolizumab vs. docetaxel among sensitivity analyses in which only the POPLAR or OAK patient cohort was included or only EGFR mutation-negative patients were included. HRs were adjusted for age, sex, race, performance status, histology, number of prior therapies, and tobacco use history. bTMB, blood-based tumor mutational burden; MSAF, maximum somatic allele frequency; OS, overall survival; PFS, progression-free survival; HR, hazard ratio; CI, confidence interval.

Among both the POPLAR and OAK subsets, the combined bTMB-MSAF classification could effectively stratify patients into subgroups with or without OS benefit from atezolizumab (Figure 4A). The findings for PFS remained robust in the OAK subset, and showed a similar trend in the POPLAR subset that had smaller sample size (Figure 4B). The results regarding OS and PFS were also consistent when only patients with EGFR mutation-negative disease were included (Figures 4A,B).

## Discussion

To the best of our knowledge, this is the first study that identifies MSAF as a predictive marker for atezolizumab vs. docetaxel among advanced NSCLC patients. The reason for the deleterious effect of high MSAF on benefits of atezolizumab remains unclear. One possible explanation is that a high MSAF could reflect a substantial tumor burden, which could result in rapid deterioration of general fitness of atezolizumab-treated patients, considering the late onset of the activity of PD-L1 blockade (17, 18). Additionally, a high MSAF may indicate a high metastatic burden, which has been found to correlate with hyperprogression after treatment with PD-1/PD-L1 inhibitors (19).

Previous studies have suggested that a low MSAF, i.e., a lower proportion of tumor-derived plasma DNA, could result in a lower detection rate of tumor somatic mutations in blood samples (8, 10). In this circumstance, a fraction of tTMB-high patients could be misclassified as bTMB-low. Supporting this speculation, our study found that atezolizumab achieved a significantly better OS compared with docetaxel in both the bTMB-high and bTMB-low subgroups. Thus, bTMB alone may be insufficient for predicting the benefit of atezolizumab. Interestingly, among patients with both a low bTMB and high MSAF, OS became numerically worse with atezolizumab than with docetaxel, suggesting that a low bTMB along with a high MSAF may indicate a true tTMB-low status, in which atezolizumab would bring no survival benefit over docetaxel. ROC analysis further demonstrated that the combined bTMB-MSAF classification outperformed bTMB and MSAF alone as a predictive biomarker for atezolizumab.

Interestingly, we observed that OS clearly favored atezolizumab in the circumstance of a high bTMB regardless of the MSAF level, and the treatment effect was comparable in the bTMB-high and MSAF-high group and the bTMB-high and MSAF-low group. A high bTMB may inform a high mutation load among the tumor, which can give rise to a high tumor neoantigen load and facilitate the development of an antitumor immune response (4, 20). Such “hot” tumor microenvironment may mitigate the detrimental effect of a high MSAF on the efficacy of atezolizumab and patient survival. Still, as the sample size of the bTMB-high and MSAF-high group was relatively small, findings from this group should be viewed with caution and further validation efforts are required.

Previous randomized studies have established anti-PD-1/PD-L1 monotherapy as the standard of care in the second- and third-line setting for advanced NSCLC (21–24). In this context, the combined bTMB-MSAF classification is particularly valuable as it can identify patients that are highly unlikely to benefit from anti-PD-1/PD-L1 monotherapy. In addition, both the bTMB and MSAF can be readily obtained from next-generation sequencing of contemporaneous blood samples, thereby supporting treatment decision-making. For patients in the bTMB-low and MSAF-high subgroup, more effective therapies are in unmet needs. Combination therapies, such as anti-PD-1/PD-L1 plus chemotherapy that has exhibited promising efficacy for advanced NSCLC (25–27), are worthy of future investigation in this subset of patients.

The strength of this study relies on the use of individual patient-level clinical data and blood-based genomic data from two randomized studies. Although a small fraction of patients was filtered out according to sample selection criteria, baseline characteristics remained balanced between treatment arms irrespective of the bTMB-MSAF status. A major limitation is that our findings require validation from external cohorts. To partially address this issue, we repeated the analyses separately in the POPLAR and OAK cohorts and obtained consistent findings. Other limitations include the retrospective, *post-hoc* nature of our analysis and the yet-to-be-determined relationship between MSAF and benefits of atezolizumab.

In summary, MSAF alone or in combination with bTMB can effectively distinguish NSCLC patients with or without OS and PFS benefit from atezolizumab compared with docetaxel. MSAF and the combined bTMB-MSAF classification may become practical, non-invasive biomarkers for atezolizumab in advanced NSCLC.

## Data Availability Statement

The datasets used and/or analyzed during the current study are available from the corresponding author upon reasonable request.

## Ethics Statement

Because the analysed data are publicly available, this study was deemed exempt from the ethical approval process and patient informed consent was waived by independent Institutional Review Boards (IRBs) of the Sun Yat-sen University Cancer Center and the Third Affiliated Hospital of Sun Yat-sen University.

## Author Contributions

YC and ZW designed this study. YC, SS, and ZW collected the data and performed statistical analysis. All authors drafted the manuscript, read, and approved the final manuscript.

### Conflict of Interest

The authors declare that the research was conducted in the absence of any commercial or financial relationships that could be construed as a potential conflict of interest.
