# Supplementary material for: Maximum Somatic Allele Frequency in Combination With Blood-Based Tumor Mutational Burden to Predict the Efficacy of Atezolizumab in Advanced Non-small Cell Lung Cancer: A Pooled Analysis of the Randomized POPLAR and OAK Studies
Source: Front Oncol. 2019 Dec 17;9:1432. doi: 10.3389/fonc.2019.01432 (PMC6929100; doi:10.3389/fonc.2019.01432)
Supplement: Supplementary file 1 [file Data_Sheet_1.PDF]

Supplemental Materials

Maximum Somatic Allele Frequency in Combination with Blood-Based Tumor Mutational Burden to Predict the Efficacy of Atezolizumab in Advanced Non-Small Cell Lung Cancer: A Pooled Analysis of the Randomized POPLAR and OAK Studies. Chen Y et al.

Supplementary Figure 1. The prognostic impact of MSAF in patients treated with docetaxel (A) and those treated with atezolizumab (B). The MSAF-low status refers a MSAF of <10.3%. HRs were adjusted for age, sex, race, performance status, histology, number of metastatic sites, number of prior therapies, and tobacco use history. Abbreviations: MSAF, maximum somatic allele frequency; HR, hazard ratio; CI, confidence interval.

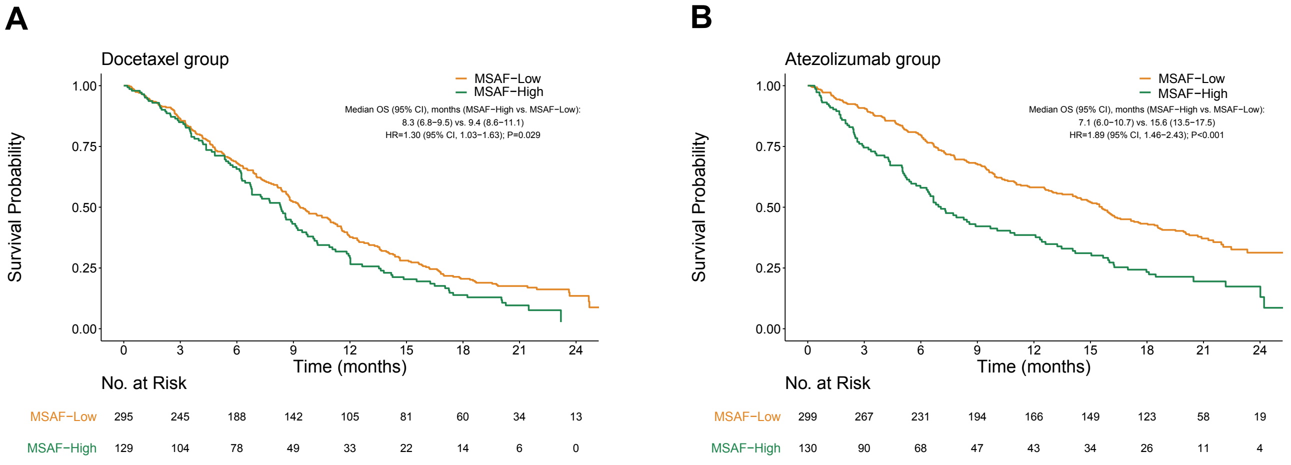

Supplementary Figure 2. The prognostic impact of the bTMB-MSAF classification (bTMB-low and MSAF-high vs. bTMB-high or MSAF-low) in patients treated with docetaxel (A) and those treated with atezolizumab (B). The bTMB-low status refers to a bTMB of <13 and the MSAF-low status refers a MSAF of <10.3%. HRs were adjusted for age, sex, race, performance status, histology, number of metastatic sites, number of prior therapies, and tobacco use history. Abbreviations: bTMB, blood-based tumor mutational burden; MSAF, maximum somatic allele frequency; HR, hazard ratio; CI, confidence interval.

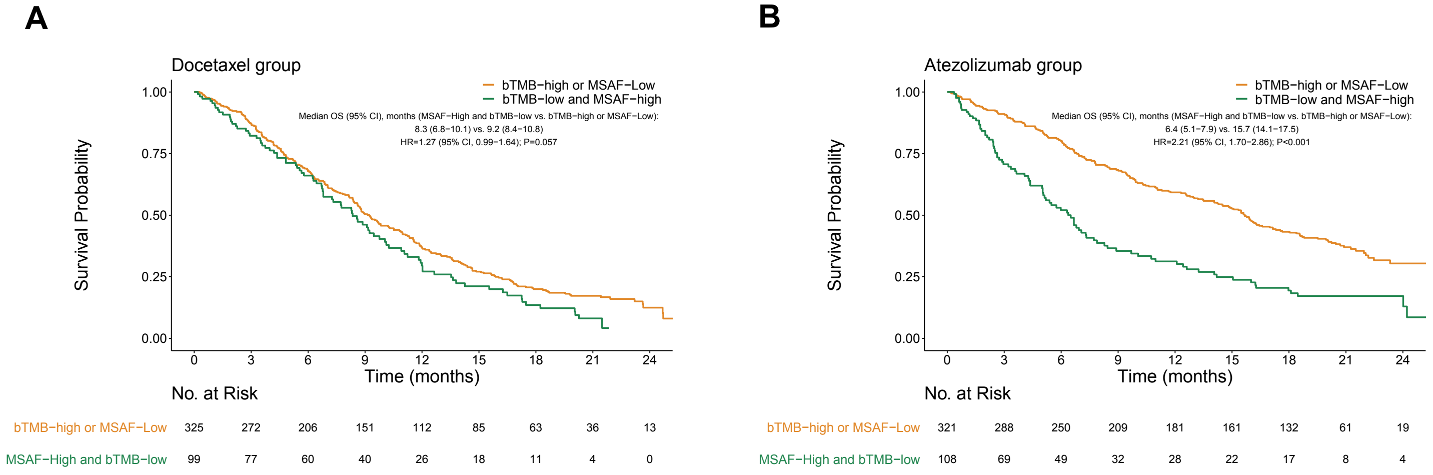

Supplementary Figure 3. Receiver operating characteristic analyses evaluating the performance of the combined bTMB-MSAF classification, as compared with that of bTMB or MSAF alone, for prediction of overall survival (A) and progression-free survival (B) in patients treated with atezolizumab. Abbreviations: bTMB, blood-based tumor mutational burden; MSAF, maximum somatic allele frequency; AUC, area under curve.

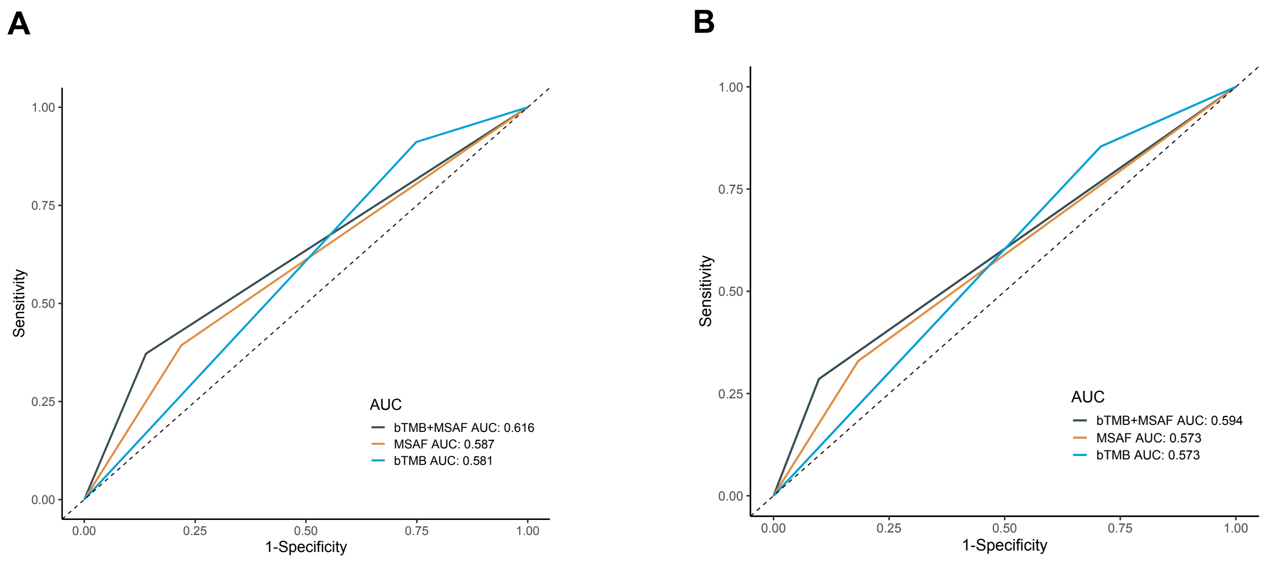

Supplementary Table 1. Patient characteristics

Variable	bTMB-high or MSAF-low		bTMB-low and MSAF-high	
	**Atezolizumab (n=325)**	**Docetaxel (n=321)**	**Atezolizumab (n=99)**	**Docetaxel (n=108)**
**Mean (SD), years**	63.4 (9.2)	63.1 (9.2)	61.9 (8.9)	62.7 (8.8)
**Race**				
**White**	238 (73.2)	226 (70.4)	74 (74.7)	78 (72.2)
**Asian**	58 (17.8)	67 (20.9)	20 (20.2)	19 (17.6)
**Other**	29 (8.9)	28 (8.7)	5 (5.1)	11 (10.2)
**Sex=Male**	186 (57.2)	196 (61.1)	66 (66.7)	79 (73.1)
**ECOG status=1**	210 (64.6)	208 (64.8)	73 (73.7)	78 (72.2)
**Histology=Squamous**	98 (30.2)	91 (28.3)	32 (32.3)	34 (31.5)
**Number of metastatic sites: >=2**	295 (90.8)	273 (85.0)	93 (93.9)	104 (96.3)
**Number of prior therapies=2**	85 (26.2)	85 (26.5)	27 (27.3)	29 (26.9)
**Tobacco use history**				
**Never**	52 (16.0)	58 (18.1)	11 (11.1)	19 (17.6)
**Current**	53 (16.3)	51 (15.9)	19 (19.2)	15 (13.9)
**Previous**	220 (67.7)	212 (66.0)	69 (69.7)	74 (68.5)
**EGFR mutation**				
**Negative**	221 (68.0)	223 (69.5)	66 (66.7)	72 (66.7)
**Positive**	28 (8.6)	29 (9.0)	5 (5.1)	6 (5.6)
**Thr790Met**	0 (0.0)	1 (0.3)	0 (0.0)	0 (0.0)
**Unknown**	76 (23.4)	68 (21.2)	28 (28.3)	30 (27.8)

Abbreviations: bTMB, blood-based tumor mutational burden; MSAF, maximum somatic allele frequency.
